# Supplementary material for: Vertical Orientation of Liquid Crystal on 4-n-Alkyloxyphenoxymethyl-Substituted Polystyrene Containing Liquid Crystal Precursor
Source: Polymers (Basel). 2021 Feb 27;13(5):736. doi: 10.3390/polym13050736 (PMC7956854; doi:10.3390/polym13050736)
Supplement: Supplementary file 1 [file polymers-13-00736-s001.pdf]

Vertical orientation of liquid crystal on

4-*n*-alkyloxyphenoxymethyl-substituted polystyrene containing liquid crystal precursor

Kyutae Seo and Hyo Kang *

BK-21 Four Graduate Program, Department of Chemical Engineering, Dong-A University,

37 Nakdong-Daero 550 Beon-gil, Saha–gu, Busan 604–714, Republic of Korea

*Corresponding author. Email: hkang@dau.ac.kr, Phone: +82 51 200 7720, Fax: +82 51 200 7728

Figure captions

Figure S1. ^{1}H nuclear magnetic resonance (NMR) spectrum of PBOP.

Figure S2. ^{1}H nuclear magnetic resonance (NMR) spectrum of PHOP.

Figure S3. ^{1}H nuclear magnetic resonance (NMR) spectrum of POOP.

Figure S4. ^{1}H nuclear magnetic resonance (NMR) spectrum of PEOP20.

Figure S5. ^{1}H nuclear magnetic resonance (NMR) spectrum of PEOP40.

Figure S6. ^{1}H nuclear magnetic resonance (NMR) spectrum of PEOP60.

Figure S7. ^{1}H nuclear magnetic resonance (NMR) spectrum of PEOP80.

Figure S8. Energy dispersive spectroscopy (EDS) mapping images of (a) the bare glass and (b)–(f) PEOP film on the glass substrate observed at different positions.

Figure S9. Energy dispersive spectroscopy (EDS) mapping images of (a)–(e) PEOP film on the glass substrate after thermal treatment at 200 °C for 10 min observed at different positions.

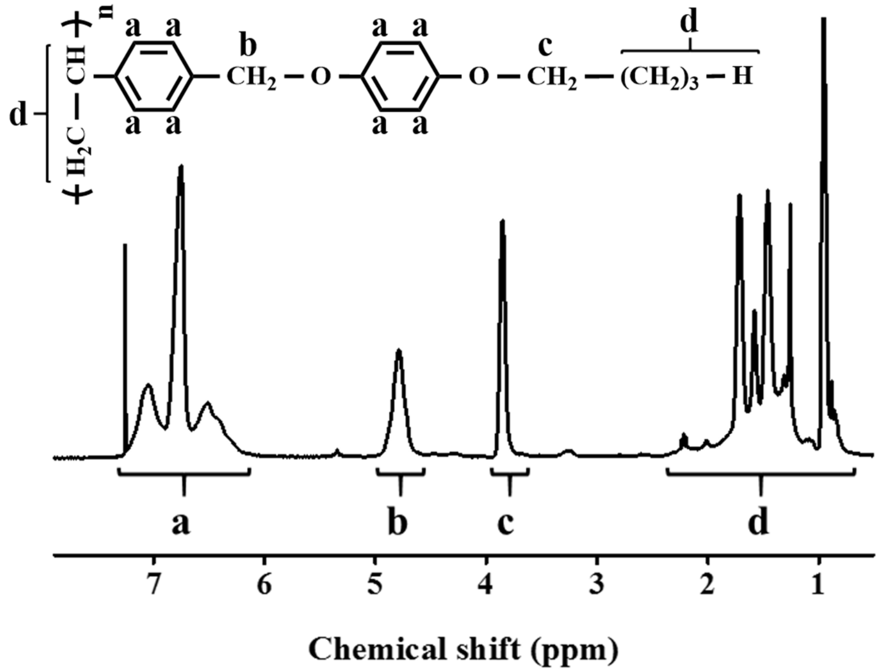

Figure S1. ^{1}H nuclear magnetic resonance (NMR) spectrum of PBOP.

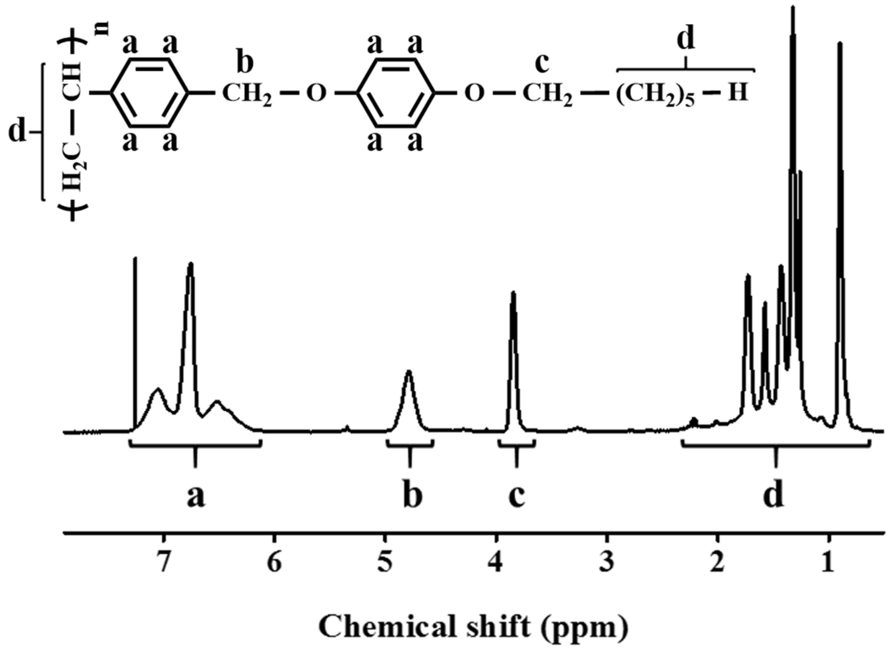

Figure S2. ^{1}H nuclear magnetic resonance (NMR) spectrum of PHOP.

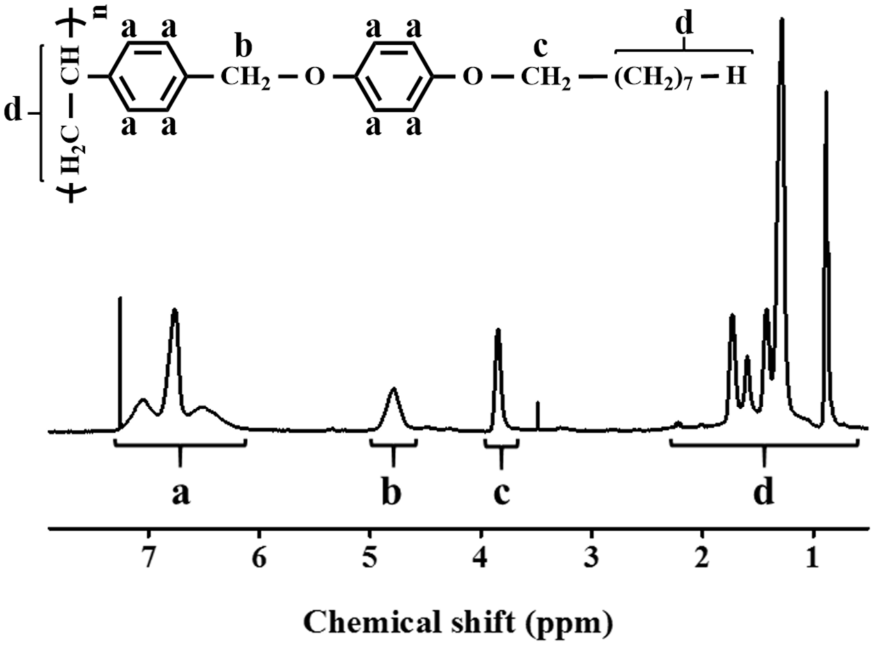

Figure S3. ^{1}H nuclear magnetic resonance (NMR) spectrum of POOP.

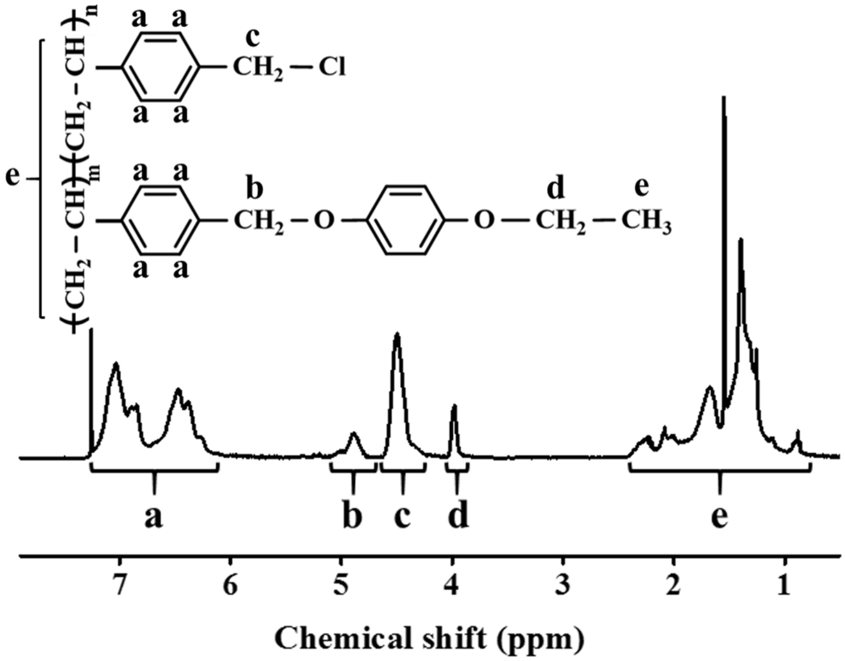

Figure S4. ^{1}H nuclear magnetic resonance (NMR) spectrum of PEOP20.

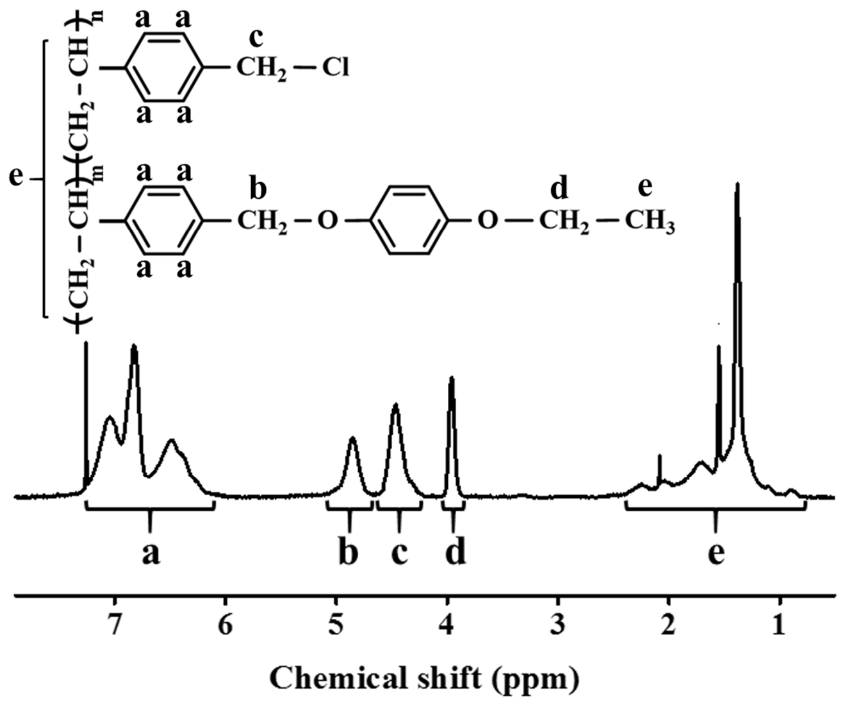

Figure S5. ^{1}H nuclear magnetic resonance (NMR) spectrum of PEOP40.

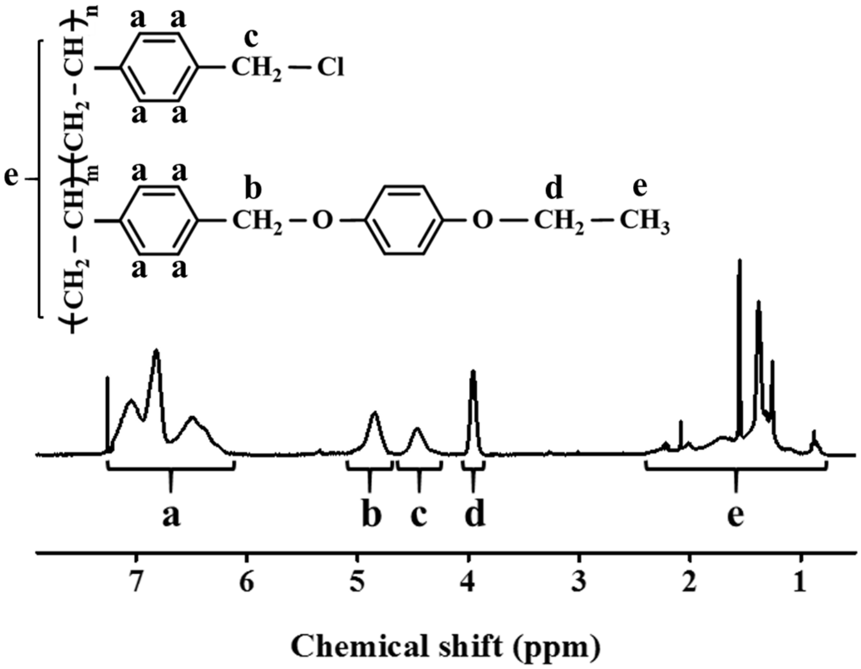

Figure S6. ^{1}H nuclear magnetic resonance (NMR) spectrum of PEOP60.

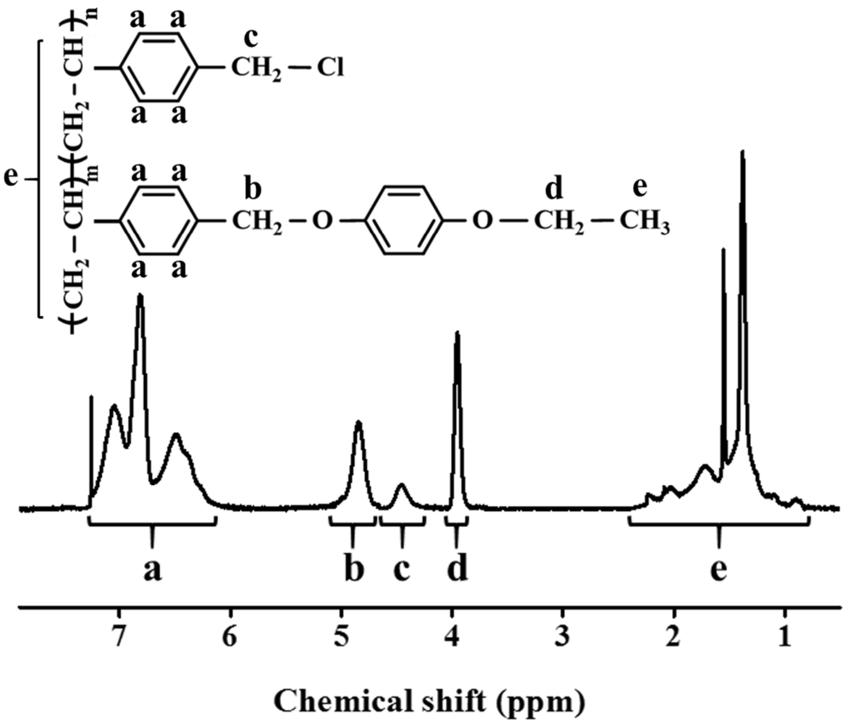

Figure S7. ^{1}H nuclear magnetic resonance (NMR) spectrum of PEOP80.

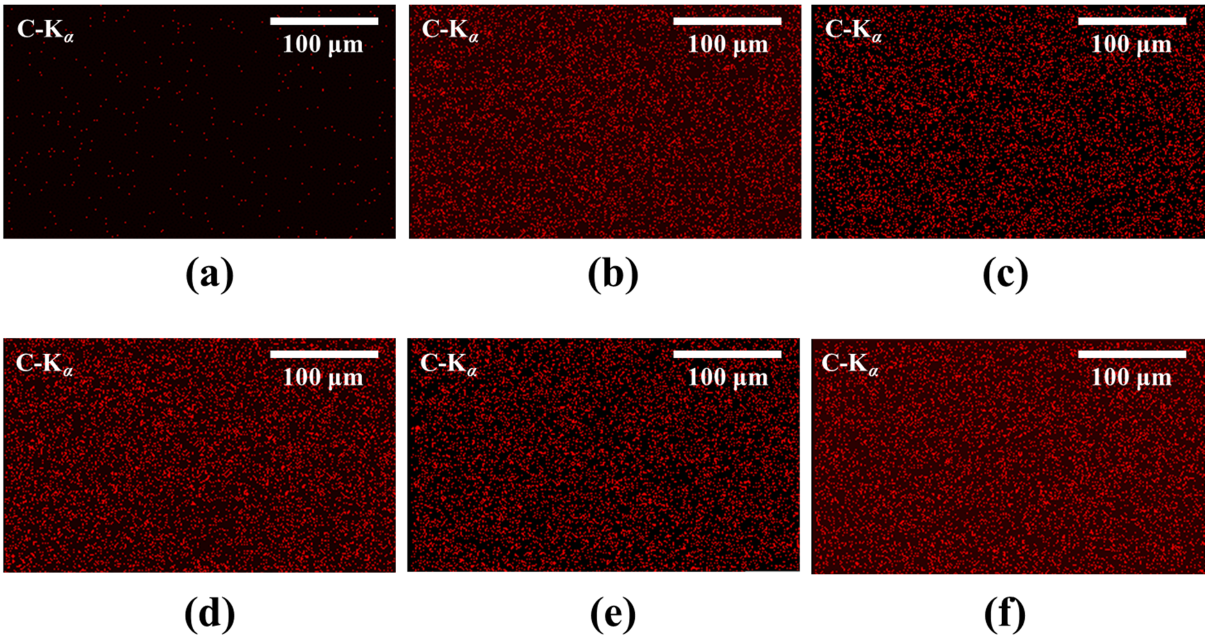

Figure S8. Energy dispersive spectroscopy (EDS) mapping images of (a) the bare glass and (b) – (f) PEOP film on the glass substrate observed at different positions.

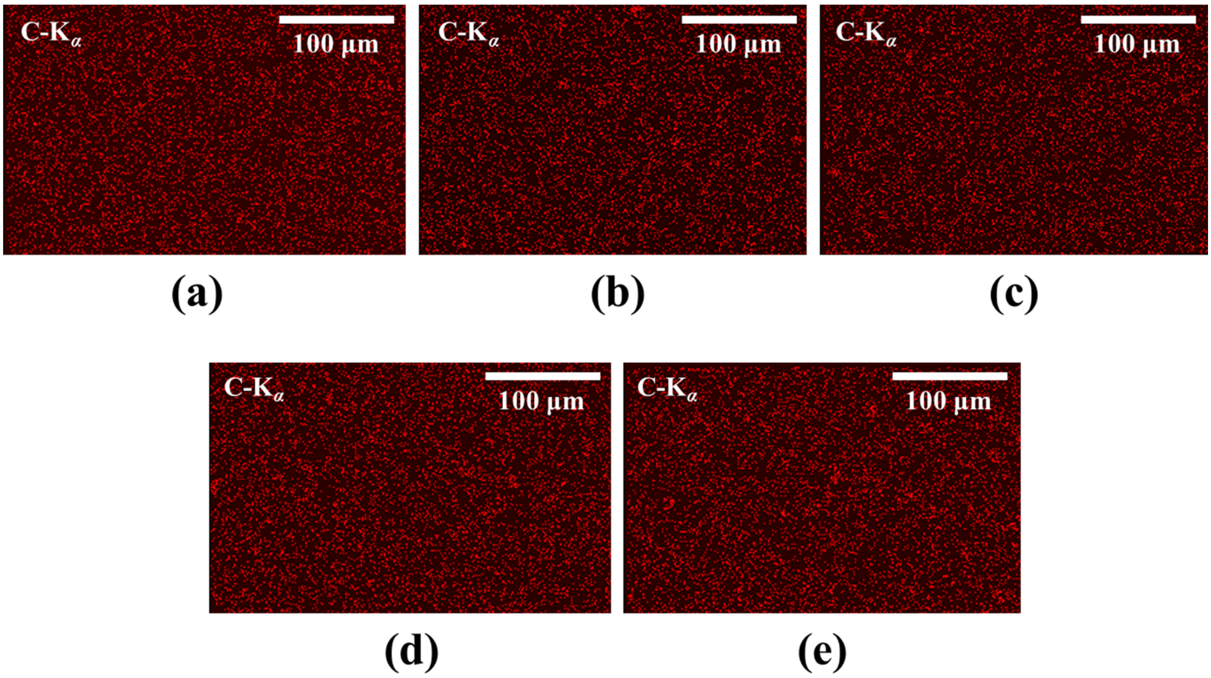

Figure S9. Energy dispersive spectroscopy (EDS) mapping images of (a) – (e) PEOP film on the glass substrate after thermal treatment at 200 °C for 10 min observed at different positions.
